# Challenges to oligonucleotides-based therapeutics for Duchenne muscular dystrophy

**DOI:** 10.1186/2044-5040-1-8

**Published:** 2011-02-09

**Authors:** Aurélie Goyenvalle, Kay E Davies

**Affiliations:** 1 MRC Functional Genomics Unit, Department of Physiology, Anatomy & Genetics, University of Oxford, Oxford, UK

## Abstract

Antisense oligonucleotides are short nucleic acids designed to bind to specific messenger RNAs in order to modulate splicing patterns or inhibit protein translation. As such, they represent promising therapeutic tools for many disorders and have been actively developed for more than 20 years as a form of molecular medicine. Although significant progress has been made in developing these agents as drugs, they are yet not recognized as effective therapeutics and several hurdles remain to be overcome. Within the last few years, however, the prospect of successful oligonucleotides-based therapies has moved a step closer, in particular for Duchenne muscular dystrophy. Clinical trials have recently been conducted for this myopathy, where exon skipping is being used to achieve therapeutic outcomes. In this review, the recent developments and clinical trials using antisense oligonucleotides for Duchenne muscular dystrophy are discussed, with emphasis on the challenges ahead for this type of therapy, especially with regards to delivery and regulatory issues.

## Review

The development of the antisense oligonucleotides (AO)-based approach started in the late 1970's when the oligonucleotides were used as tools to downregulate the expression of specific genes [1]. The strategy was intuitive: oligonucleotides could be designed to hybridize with a specific mRNA target and mediate its destruction by RNaseH, an enzyme that destroys the RNA in a DNA/RNA complex. Attention rapidly increased with the development of antisense molecules for manipulation of alternative splicing. In this context, oligonucleotides can be used to modulate the ratio of splicing variants or correct splicing defects, which opened far-reaching implications in the treatment of a variety of diseases. The requirements for oligonucleotides that alter splicing are different from those for oligonucleotides used to achieve downregulation. In particular, they must not activate RNaseH, which would destroy the pre-mRNA before it could be spliced. They must also access their target pre-mRNAs within the nuclei of cells to efficiently compete with splicing factors. Several types of modified synthetic oligonucleotides fit these criteria. Among these, oligonucleotides with modifications to the 2' position, such as 2'-O-methyl (2'OMe), 2'-O-methoxyethyl (2'O-MOE) and 2'-O-aminopropyl, are RNaseH inactive and display higher nuclease resistance and affinity for target sequences than their 2'-deoxy counterparts. Similar characteristics are found in oligonucleotides with backbones based on morpholino, peptide nucleic acid (PNA), locked nucleic acid (LNA), phosphoramidate and methyl-phophonate derivatives. These advances in the development of antisense chemistries have led to numerous studies investigating the therapeutic potential of antisense technology (for review see [2]). However, despite early promise, the therapeutic application of AO has proved to be difficult and has been very slow entering the market and standard of care. Only a single AO compound has been approved by the Food and Drug Administration (FDA) so far. Indeed, no other AO has won marketing approval since Vitravene (Fomivirsen), developed by ISIS Therapeuticals was approved in 1998 for use against cytomegalovirus-induced retinitis by intravitreus injection [3]. Among the hurdles that have slowed the progress of AO drugs into the clinical area are off-target toxic effects and low efficacy partly due to delivery difficulty.

Recent developments are achieving success overcoming some of these obstacles. In particular, the use of AO in clinical trials for Duchenne muscular dystrophy (DMD) has recently demonstrated very encouraging results. The remainder of this review focuses on this application. We will first review the principle of this approach and the clinical data from these initial trials and we will then discuss the promises and challenges of AO based therapy for DMD, focusing on systemic delivery and regulatory issue.

### DMD background and rationale of exon skipping therapy

Duchenne muscular dystrophy (DMD) is a lethal X-linked progressive muscle-wasting disease caused by mutations, typically large deletions in the DMD gene, the largest gene in the human genome [4]. Most mutations, including deletions (approximately 65%), duplications, point mutations or other small gene rearrangements disrupt the open reading frame, leading to aberrant translation and, therefore, to the absence of the essential muscle protein dystrophin. Dystrophin is localized at the sarcolemma of the muscle fiber and forms a dystrophin glycoprotein complex (DGC) with dystroglycan, sarcoglycan, and syntrophin/dystrobrevin complexes. The DGC provides a mechanical and signalling link between the actin cytoskeleton and the extracellular matrix [5]. The absence of dystrophin leads to recurrent muscle fiber damage during contraction and muscle fibers are eventually replaced by adipose and fibrotic tissue. Patients with DMD suffer from progressive loss of muscle function which generally leads to wheelchair dependency by the age of 13 and premature death, mostly before the age of 30 [6].

**Figure 1:**
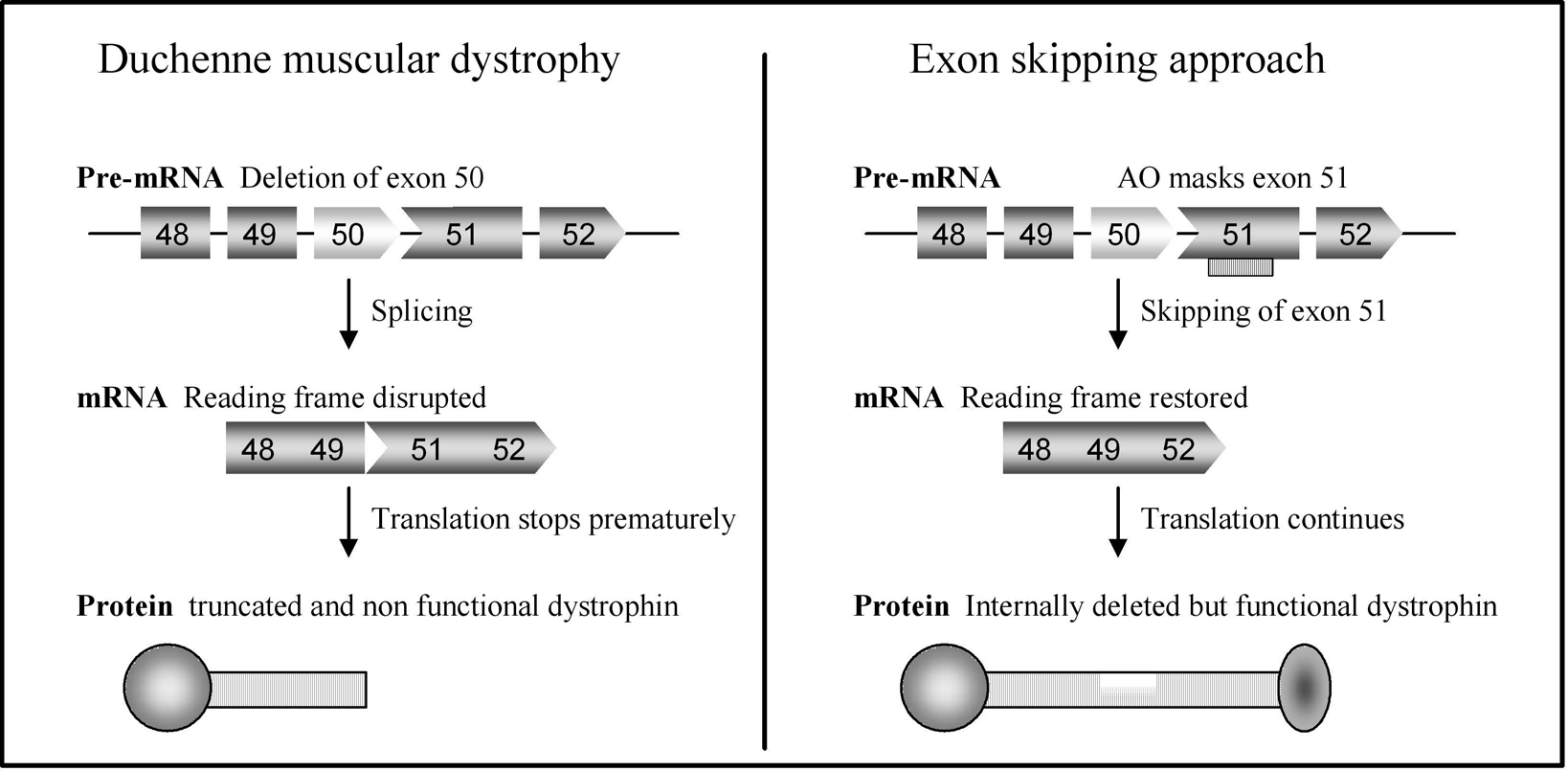
**Antisense-mediated exon skipping rationale for DMD**. Patients with Duchenne muscular dystrophy have mutations which disrupt the open-reading frame of the dystrophin pre-mRNA. In this example, exon 50 is deleted, creating an out-of-frame mRNA and leading to the synthesis of a truncated non-functional or unstable dystrophin (left panel). An antisense oligonucleotide directed against exon 51 can induce effective skipping of exon 51 and restore the open reading frame, therefore generating an internally deleted but partly functional dystrophin (right panel).

Interestingly, the allelic disease Becker muscular dystrophy (BMD), which results in a much milder phenotype, is mainly caused by mutations maintaining the open reading frame and allowing the production of a partially deleted but functional dystrophin [7]. Antisense-mediated exon-skipping strategies for DMD aim to remove the mutated exon alone or together with additional exons to restore the reading frame and consequently induce the expression of "BMD-like" shortened forms of dystrophin retaining crucial functions (Figure 1). Although the exon-skipping approach appears to be applicable to a large proportion of patients (possibly up to approximately 83% of all DMD patients [8], one should keep in mind that this will not offer a definite cure but an improvement towards a BMD-like phenotype depending on the functionality of the restored dystrophin.

**Figure 2:**
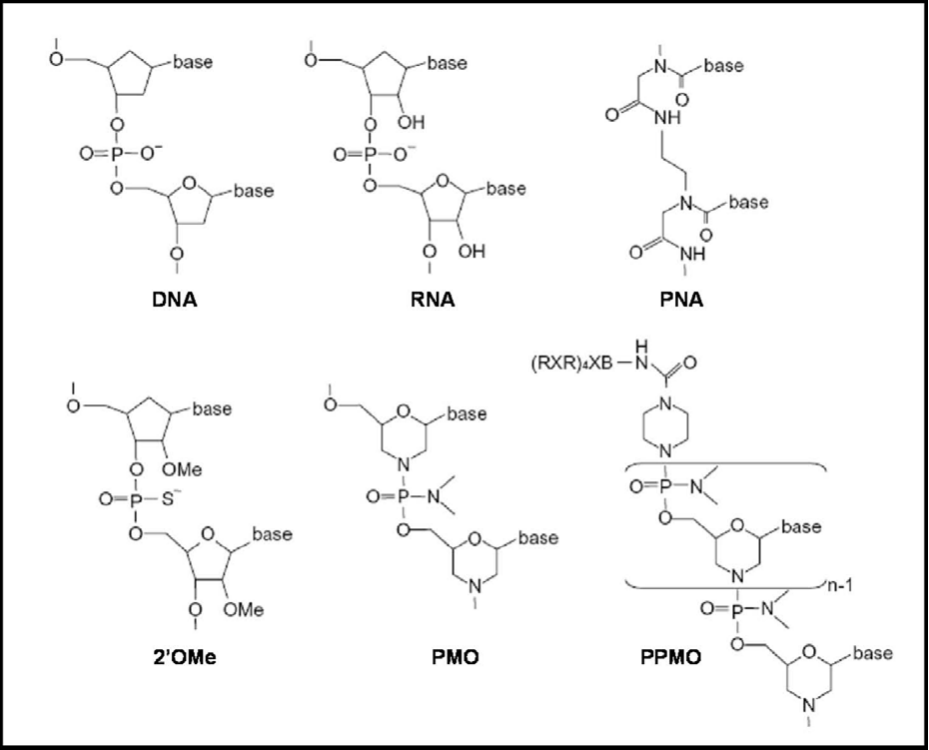
**Chemistries used for the exon skipping approach**. Artificially developed AO such as peptide nucleic acid (PNA), 2'O-Methyl-phosphorothiate-antisense oligonucleotides (2'OMeAO), phosphorodiamidate morpholino oligomer (PMO) and peptide conjugated PMO (PPMO) are shown for comparison with DNA and RNA. PNA's backbone is composed of repeating N-(2-aminoethyl)-glycine units linked by peptide bonds, PMO have a morpholine ring instead of the deoxyribose ring in DNA or ribose ring in RNA and 2'OMeAO are similar to RNA but methylated at the 2'-OH position of the ribose ring.

The principle of the exon-skipping therapy for DMD has first been demonstrated by Pramono *et al. *in 1996 in lymphoblastoid cells and by Dunckley *et al. *in 1998 in cultured mouse cells *in vitro *[9,10]. Since then, numerous *in vivo *studies have provided pre-clinical evidence for the therapeutic potential of an antisense strategy for DMD in several animal models. In particular, the *mdx *mouse model, which harbors a nonsense mutation in exon 23, has been used extensively to test efficacy of the AO approach using various oligonucleotides chemistries such as 2'OMe [11], phosphorodiamidate morpholino oligomers (PMO) [12,13], LNA or PNA [14,15] (Figure 2). Intramuscular and systemic injections in canine models of the disease have also demonstrated restoration of dystrophin expression associated with functional benefits [16].

Following the very encouraging results obtained on these animal models, groups both in the Netherlands and in the UK have worked towards clinical evaluation of the antisense mediated exon-skipping in DMD patients.

### Phase I clinical trials provide proof of principle of oligonucleotide-based therapy for DMD

The clinical application of antisense-mediated exon-skipping for DMD raises several considerations. Among these, the genetic heterogeneity of DMD patients means that this approach is mutation-specific and as such an example of personalized medicine. According to the Leiden muscular dystrophy database, exon-skipping is potentially applicable to approximately 83% of all DMD patients if single and double exon-skipping of deletions, small mutations and duplications can be achieved [8]. Fortunately, the majority of deletions clusters into hotspot regions between exons 43 and 53, suggesting that skipping of the same group of exons is applicable to large groups of patients. The most notable example is exon 51 skipping, which is applicable to 13% of all patients and has for that reason been targeted for both phase I clinical trials.

The Dutch group together with the RNA therapy company Prosensa selected a 20-mer antisense oligonucleotide of 2'OMe phosphorothiate RNA chemistry targeting exon 51 (PRO051). Four DMD patients were injected locally in the tibialis anterior muscle with a single dose of 0.8 mg PRO051. Four weeks after the injection, a small biopsy was analyzed for each patient and revealed a restoration of dystrophin in the vast majority of muscle fibers at levels varying between 17 and 35%, in the absence of treatment related adverse effect [17]. The UK team in collaboration with AVI Biopharma selected a PMO antisense oligonucleotide, based on pre-clinical studies with this backbone chemistry [12,13,18]. The 30-mer PMO optimized to skip exon 51 (AVI-4658) [19] was injected unilaterally into the extensor digitorum brevis muscles of seven patients, in a single-blind, dose-escalation protocol that included a placebo control administered to the contralateral extensor digitorum brevis muscle. Results from this trial demonstrated that PMO oligonucleotides were well tolerated by all patients and that dystrophin protein were expressed at up to 42% of normal levels in dystrophin positive fibers of patients treated with the higher dose of 0.9 mg [20].

**Figure 3:**
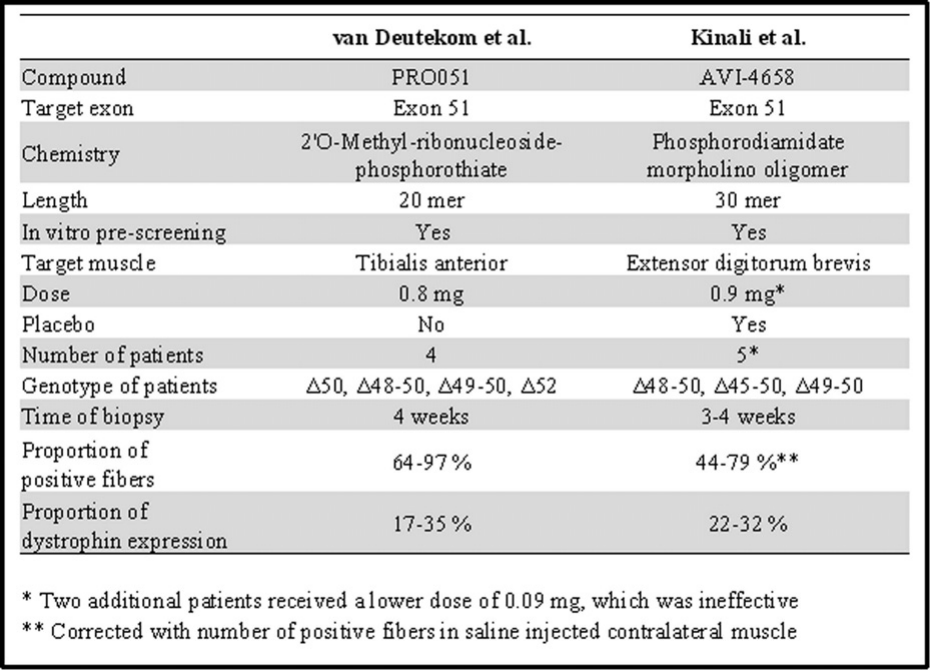
**Comparison of the two clinical trials reporting intramuscular injection of AO in patients with DMD **[17,20].

Although there are similarities and differences between the two studies (summarized in Figure 3), they both report unequivocal expression of dystrophin at similar concentrations without any drug-related adverse effect with either chemistry. These very encouraging results confirmed the proof of the principle of the antisense oligonucleotide based therapy for DMD. However, they represent only a first step as intramuscular injection of each individual muscle is not feasible. The next step which both groups are currently undertaking is to deliver the antisense oligonucleotide systemically.

### Systemic delivery of Antisense oligonucleotides for DMD

The systemic delivery of AO for the treatment of DMD has been demonstrated in mouse and dog models without safety issues. Intramuscular clinical trials performed with either a 2'OMe (PRO051) or a PMO (AVI-4658) provided a proof-of-principle efficacy study in man. Both studies have been followed by repeated systemic administration studies. The Dutch consortium recently completed a phase I/IIa trial involving four groups of DMD boys receiving escalating doses of 0.5, 2.0, 4.0 and 6.0 mg/kg of 2'OMe weekly for five weeks. While the results of this study has not yet been published, Dr Goemans reported at the World Muscle Society (WMS) meeting in 2009 that PRO051 was well tolerated in each DMD patient and that novel dystrophin expression was observed [21]. All boys who participated in this study entered an open label extension study, receiving weekly subcutaneous injections of 6 mg/kg regardless of earlier dose. Twenty-four-week follow-up data presented at the WMS meeting in 2010 reported that this treatment was generally well tolerated over the 24-week period and that encouraging gains in the six-minute walk test were observed in some boys [22]. Encouraging results have also been announced by the UK consortium and AVI Biopharma who have completed their systemic study enrolling 19 patients receiving weekly IV administrations of AVI-4658 (six cohorts receiving 0.5, 1.0, 2.0, 4.0, 10.0 or 20.0 mg/kg). Dr Shrewsbury reported at the WMS meeting in 2010 that the study drug was well tolerated and that exon 51 skipping was detected in patients at the 2 mg/kg dose and above, giving rise to expression of dystrophin [23]. These preliminary results sound promising and raise expectations to a high level for the systemic treatment of DMD. However, one should not forget that many challenges remain, especially regarding the delivery to all affected tissues in DMD.

### Current challenges regarding delivery

Although results from initial trials appear very encouraging, there are several issues that pose challenges for the use of AO, either 2'OMe or PMO- as effective and affordable drugs for DMD. The first obstacle with both chemistries remains the poor cellular uptake and relative rapid clearance from the circulation, which require repeated administration to achieve some therapeutic efficacy. Experiments in animal models demonstrated that large doses ranging from 100 mg/kg/wk in mouse [24,25] to 200 mg/kg/wk in dogs [16] over several weeks were required for functional improvement. The fact that both animal models required large doses, despite their difference in body surface area, suggests that similar dose range might be required to achieve efficacy in humans. If so, the cost of an AO drug, type PMO for example, for life-long treatment will probably be prohibitive for many patients.

A second major hurdle with the systemic administration of AO is the high variability in exon-skipping efficiency among muscle types. Again data collected from mice and dog studies have shown that some muscles respond better than others. Repeated intravenous injections of 100 mg/kg of PMOE23 to *mdx *mice resulted in higher restoration of dystrophin in quadriceps, abdominals, intercostals and gastrocnemii muscles compared to diaphragm, biceps, triceps and tibialis anterior, for example [24]. Even within the most responsive muscles, dystrophin was not uniformly expressed with detection of patches of dystrophin-positive fibers and patches of dystrophin-negative fibers. This may be because the uptake of AO is restricted to leaky muscle fibers, indicating that AO's efficacy in humans will depend on the number of leaky muscle fibers.

An additional problem compromising the therapeutic potential of AO-mediated exon-skipping for DMD at the moment is that both AO chemistries have shown very little efficacy in the cardiac muscle [24,26]. A more recent study showed that even higher doses (nine doses of 100 mg/kg) induced only very low level (1 to 2%) of dystrophin expression in the heart with both 2'OMe and PMO [25]. Only when used in huge amounts such as 300 mg/kg and 3,000 mg/kg, could PMOE23 restore about 5% and 30% respectively of wild-type dystrophin level in the heart of *mdx *mice [27]. Such extreme doses would be unsustainable for repeated administration and the long-term treatment required for DMD. Clinically, cardiomyopathy is the second leading cause of death in patients with DMD in countries where ventilator therapy has been introduced, accounting for 10 to 40% of deaths in DMD populations [4], which implies a clear need for a cardiac dystrophin correction. The reasons for the low efficiency of cardiac dystrophin restoration are unclear, but are probably related to the poor ability of unmodified oligonucleotides to penetrate the heart.

Recent developments using cell penetrating peptides (CPP)-conjugated PMO (PPMO) have addressed most of these delivery issues and could, therefore, represent an effective strategy to reduce dose level and dose frequency, as well as delivering the AO to non-leaky fibers and the heart [27-31].

### Challenges and promises of PPMO for DMD

Since the first study reported the enhanced PMO uptake mediated by a cell penetrating peptide, resulting in widespread restoration of dystrophin in *mdx *mice in 2007 [13], PPMO have gained attention for the systemic treatment of DMD. Many laboratories have demonstrated uniform and high levels of dystrophin expression through the whole body using much lower dose of PPMO (ranging from 6 to 25 mg/kg) compared to PMO (around 100 mg/kg) [28,32,33]. PPMO are much more effective than PMO because of the ability of CPP to facilitate the internalization of PMO through an active process, unlike the passive diffusion process for PMO. PPMO are internalized by nearly all muscle cells, and, therefore, not restricted to leaky fibers as are PMOs [31]. Moreover, evidence of significantly restored cardiac dystrophin has been demonstrated in PPMO-treated mice [27-30]. A single intravenous injection of 30 mg/kg of PPMO indeed restored dystrophin to almost normal levels in the cardiac and skeletal muscles of *mdx *mice, which leads to an increase in muscle strength and, more importantly, improvement of cardiac function [30].

Taken all together, these studies indicate that PPMO can be used at a much lower dose than PMO and can achieve more widespread restoration of dystrophin throughout the whole body's muscle including the heart. These characteristics would qualify PPMO as an ideal candidate for the systemic treatment of DMD compared to unconjugated AO. However, the toxicity of current PPMO chemistry poses a challenge for determination of an effective and safe regimen in man. One PPMO targeting human exon 50 (AVI-5038) is currently in pre-clinical development for DMD and has been tested in the cynomolgus monkey [31]. This PPMO was found to cause mild tubular degeneration in the kidneys of monkeys injected weekly with 9 mg/kg for four weeks, although the same peptide conjugated to PMOE23 did not exhibit any toxic effect in the kidneys of *mdx *mice treated with higher doses (30 mg/kg biweekly for three months) [30]. This indicates that monkeys are more sensitive to PPMO-related toxicity than mice. The nature of the toxicity is not well understood, but it is likely to be due to the cationic nature of the peptide. A dose threshold for the toxicity seems to exist, which level depends on the amino acid composition of the peptide [31]. Another major concern that arises with the use of peptides as delivery enhancers is the immune response they might elicit. It is, therefore, extremely important to monitor such a response in animal models like Wu *et al. *did [30]. The use of unnatural amino acids in some of these peptide sequences most likely contributes to the lack of immunogenicity. However, the principle of precaution together with the fact that immunogenicity varies considerably between species would argue for longer term studies in other species than mice. These pre-clinical data provide valuable information and emphasize the difficulty in predicting exon-skipping efficacy/toxicity across species and to plan efficient yet safe escalation in human patients.

### Current regulatory challenges of personalized medicine

Despite the very promising results of the initial trials targeting exon 51, the clinical applicability of the AO-mediated exon-skipping approach for DMD still faces a major hurdle regarding regulatory approval. The sequence specific nature of the strategy has implications for future personalised medicine. Although skipping of exon 51 is applicable to a large group of DMD patients (13%), it will not benefit the other 87% and, therefore, other AO need to be developed to target other dystrophin exons [8]. From the Leiden muscular dystrophy database, it has been estimated that skipping 10 exons might be beneficial in up to 40% of all patients and that this could be increased to 83% if single and double skipping of point mutations, duplications and small mutations could be achieved [8]. One can easily envision that numerous specific sequences will be required to effectively treat such a large proportion of patients. If each AO is considered a new drug, which is the current FDA regulation, then each of them will have to go through the expensive and lengthy clinical trial stages. On top of being an insuperable barrier in terms of money and time, it might be very problematic to find enough patients to even perform clinical trials. Some AO may be applicable to a very restricted number of patients, such as those targeting exons 71, 72, 75, 77 or 78 (representing 0.02% of all mutations) [8]. A practical resolution of this problem would be to consider AOs (PMO or 2'OMe) as one drug, even if their sequences are different. Therefore, the realization of the clinical applicability of AO-based exon-skipping as a treatment for DMD might lie in the approval of antisense sequences as a class of drugs. This type of approval would be a first for the FDA, but the prospect of personalized molecular medicine might justify such a change in approach.

## Conclusions

Within the last few years, oligonucleotides-based therapeutics have moved a step closer to clinical applicability, especially for DMD. The first clinical trials in DMD patients have demonstrated the proof of the principle of exon-skipping in man and shown very encouraging results. Although this type of therapy faces some challenges, in particular regarding delivery to all affected tissues, recent pre-clinical work using cell-penetrating peptide suggests that solutions are close at hand. Progress made in the context of DMD may also impact the development of experimental therapies for many other disorders, such as spinal muscular atrophy (SMA) or myotonic dystrophy for which AO-mediated approaches have been investigated.

Finally, the success of oligonucleotides-based therapies will require close cooperation with regulatory agencies both in Europe and in the USA, which need to look at such "personalized medicine" in a new way to allow safe and cost-effective testing for rapid clinical development.

## Abbreviations

AO: Antisense Oligonucleotide; BMD: Becker Muscular Dystrophy; CPP: Cell Penetrating Peptide; DMD: Duchenne Muscular Dystrophy; IV: Intravenous; LNA: Locked Nucleic Acid; PMO: Phosphoradiamidate Morpholino Oligomer; PNA: Peptide Nucleic Acid; PPMO: Peptide Conjugated PMO; SMA: Spinal Muscular Atrophy; WMS: World Muscle Society; 2'OMe: 2'-O-Methyl; 2'O-MOE: 2'-O-Methoxyethyl

## Competing interests

The authors declare that they have no competing interests.

## Authors' contributions

AG and KED wrote the manuscript. All authors read and approved the final manuscript.
